# Murine IL-17^+^ Vγ4 T lymphocytes accumulate in the lungs and play a protective role during severe sepsis

**DOI:** 10.1186/s12865-015-0098-8

**Published:** 2015-06-03

**Authors:** Maria Fernanda de Souza Costa, Catarina Bastos Trigo de Negreiros, Victor Ugarte Bornstein, Richard Hemmi Valente, José Mengel, Maria das Graças Henriques, Claudia Farias Benjamim, Carmen Penido

**Affiliations:** Laboratório de Farmacologia Aplicada, Departamento de Farmacologia, Farmanguinhos, Fundação Oswaldo Cruz, Rua Sizenando Nabuco 100, Manguinhos, Rio de Janeiro, RJ CEP 21041-250 Brazil; Centro de Desenvolvimento Tecnológico em Saúde, Instituto Nacional de Ciência e Tecnologia de Inovação em Doenças Negligenciadas (INCT-IDN), Fundação Oswaldo Cruz, Rio de Janeiro, Brazil; Laboratório de Toxinologia, Instituto Oswaldo Cruz, Fundação Oswaldo Cruz, Rio de Janeiro, Brazil; Laboratório de Imunologia, Faculdade de Medicina de Petrópolis, Petrópolis, Rio de Janeiro, Brazil; Instituto Oswaldo Cruz, Fundação Oswaldo Cruz, Rio de Janeiro, Brazil; Laboratório de Inflamação, Estresse Oxidativo e Câncer, Centro de Ciências da Saúde, Instituto de Ciências Biomédicas, Universidade Federal do Rio de Janeiro, Rio de Janeiro, Brazil; Mount Sinai School of Medicine, New York City, USA

**Keywords:** γδ T cell, Interleukin-17, Chemokines, Sepsis

## Abstract

**Background:**

Lung inflammation is a major consequence of the systemic inflammatory response caused by severe sepsis. Increased migration of γδ T lymphocytes into the lungs has been previously demonstrated during experimental sepsis; however, the involvement of the γδ T cell subtype Vγ4 has not been previously described.

**Methods:**

Severe sepsis was induced by cecal ligation and puncture (CLP; 9 punctures, 21G needle) in male C57BL/6 mice. γδ and Vγ4 T lymphocyte depletion was performed by 3A10 and UC3-10A6 mAb i.p. administration, respectively. Lung infiltrating T lymphocytes, IL-17 production and mortality rate were evaluated.

**Results:**

Severe sepsis induced by CLP in C57BL/6 mice led to an intense lung inflammatory response, marked by the accumulation of γδ T lymphocytes (comprising the Vγ4 subtype). γδ T lymphocytes present in the lungs of CLP mice were likely to be originated from peripheral lymphoid organs and migrated towards CCL2, CCL3 and CCL5, which were highly produced in response to CLP-induced sepsis. Increased expression of CD25 by Vγ4 T lymphocytes was observed in spleen earlier than that by αβ T cells, suggesting the early activation of Vγ4 T cells. The Vγ4 T lymphocyte subset predominated among the IL-17^+^ cell populations present in the lungs of CLP mice (unlike Vγ1 and αβ T lymphocytes) and was strongly biased toward IL-17 rather than toward IFN-γ production. Accordingly, the *in vivo* administration of anti-Vγ4 mAb abrogated CLP-induced IL-17 production in mouse lungs. Furthermore, anti-Vγ4 mAb treatment accelerated mortality rate in severe septic mice, demonstrating that Vγ4 T lymphocyte play a beneficial role in host defense.

**Conclusions:**

Overall, our findings provide evidence that early-activated Vγ4 T lymphocytes are the main responsible cells for IL-17 production in inflamed lungs during the course of sepsis and delay mortality of septic mice.

**Electronic supplementary material:**

The online version of this article (doi:10.1186/s12865-015-0098-8) contains supplementary material, which is available to authorized users.

## Background

Mortality induced by sepsis is highly associated with secondary acute lung injury. Systemic inflammation during sepsis leads to acute respiratory distress syndrome (ARDS) caused by an exacerbated response of the immune system to bacteria and their products [1–4]. Indeed, mice subjected to experimental model of sepsis induced by cecal ligation and puncture (CLP) show deregulation in pulmonary immune response, marked by cytokine storm and intense accumulation of activated leukocytes in lung tissue, including T lymphocytes [5–8].

γδ T lymphocytes are unconventional lymphocytes that have antigen recognition properties fundamentally different from those of αβ T lymphocytes, and are comprised by distinct functional subsets, defined by the differential usage of Vγ and Vδ gene repertoire [9, 10]. The Vγ4 T lymphocyte subset is highly associated with lung immune surveillance and increases in number in mouse lungs at early time points during bacterial infections [10–13]. Increased migration of γδ T cells into the lungs has been previously demonstrated during experimental sepsis; however, the identification of γδ T cell subtypes has not been previously described [7, 8, 14].

The migration of γδ T lymphocytes is largely dictated by the activation of chemokine receptors by their counterpart ligands, among which members from both CC and CXC subfamilies play compelling roles [15–17]. Once at the infection site, these cells can rapidly respond to microbial antigens via innate surface receptors [18–21], producing high amounts of interferon (IFN)-γ and interleukin (IL)-17, which are signature cytokines produced by specific subsets of γδ T cells [22–26]. Vγ4 T lymphocytes represent one of the major subsets that produce IL-17 in different experimental models [27–30].

γδ T lymphocytes have been shown to play divergent roles in mouse models of sepsis [8, 14, 31–34]. The protective role of γδ T lymphocytes during experimental sepsis has been attributed to the production of IL-17, a cytokine that triggers neutrophil recruitment and improves bacterial clearance [33, 35–37]. Furthermore, the accumulation of activated γδ T lymphocytes in the lungs of CLP mice has been correlated with beneficial outcome of septic mice [8, 14]. Here we show that during the course of experimental severe sepsis, Vγ4 T lymphocytes migrate into injured lungs of CLP mice and exert a protective role via the production of IL-17.

## Results

### Activated γδ T lymphocytes accumulate in mouse lungs during severe sepsis

**Fig. 1:**
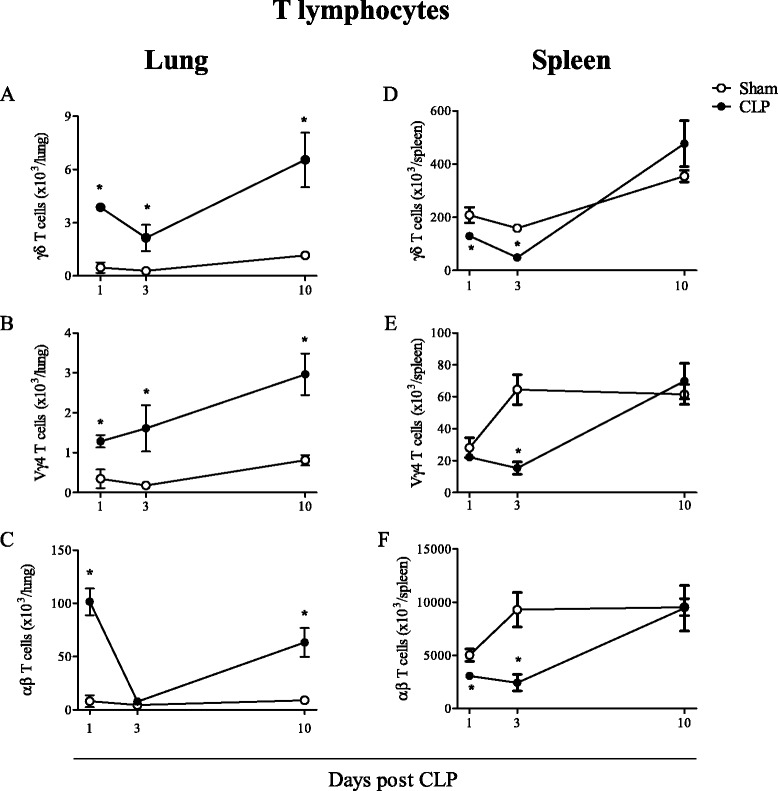
γδ T lymphocytes accumulate in mouse lungs and spleen after CLP. γδ, Vγ4 and αβ T lymphocyte numbers in C57BL/6 mouse lungs (**a**–**c**) and spleen (**d**–**f**) 1, 3 and 10 days after CLP. Results are expressed as mean ± SEM from at least five animals per group out of three different experiments. Statistical differences between the CLP and sham group (p < 0.05) are indicated by (*). The gates were set according to IgG isotype staining

The induction of severe sepsis triggered an intense inflammatory response in mouse lungs, marked by a significant increase of γδ and αβ T lymphocyte numbers observed from 1 to 10 days after the surgery (Fig. 1a-c). The γδ T cell subtype Vγ4 also infiltrated into the lungs of CLP-induced mice and, differently from those of αβ and γδ T lymphocytes, did not decrease in numbers at day 3 post-surgery. Both γδ and αβ T lymphocyte numbers were decreased in mouse spleen from 1–3 days after CLP, returning to control (sham-operated mice) levels within 10 days (Fig. 1d-f), suggesting that T cells found in the lungs egress from secondary lymphoid organs.

**Fig. 2:**
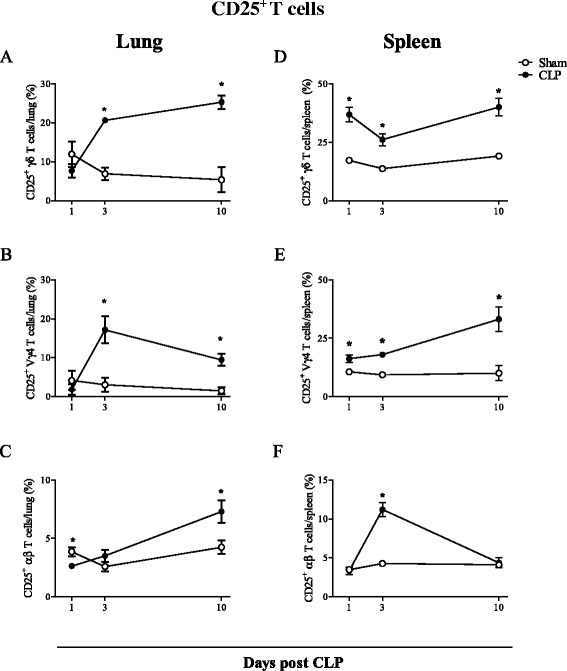
Activated γδ T lymphocytes accumulate in mouse lungs and spleen after CLP. Percentages of CD25^+^ cells among γδ, Vγ4 and αβ T lymphocyte populations recovered from mouse lungs (**a**–**c**) and spleen (**d**–**f**) 1, 3 and 10 days after CLP. Results are expressed as mean ± SEM from at least five animals per group out of three different experiments. Statistical differences between the CLP and sham group (p < 0.05) are indicated by (*). The gates were set according to IgG isotype staining

The analysis of activation marker expression revealed that the presence of CD25^+^ T lymphocytes in the lungs and spleen of CLP mice was more expressive among γδ rather than among αβ T cell population. The percentages of γδ (and Vγ4^+^) T lymphocytes expressing CD25 were increased in the lungs of CLP mice at day 3 post CLP and persisted elevated until 10 days after surgery, when the number of αβ T lymphocyte also increased (Fig. 2a-c). In spleens, γδ and Vγ4 T lymphocytes were constantly activated during the course of experimental severe sepsis, whereas CD25^+^ αβ T lymphocytes were only elevated at day 3 after CLP (Fig. 2d-f).

### γδ T lymphocytes migrate from spleen into the lungs of CLP-operated recipient mice coordinated by lymphotactic chemokines

**Fig. 3:**
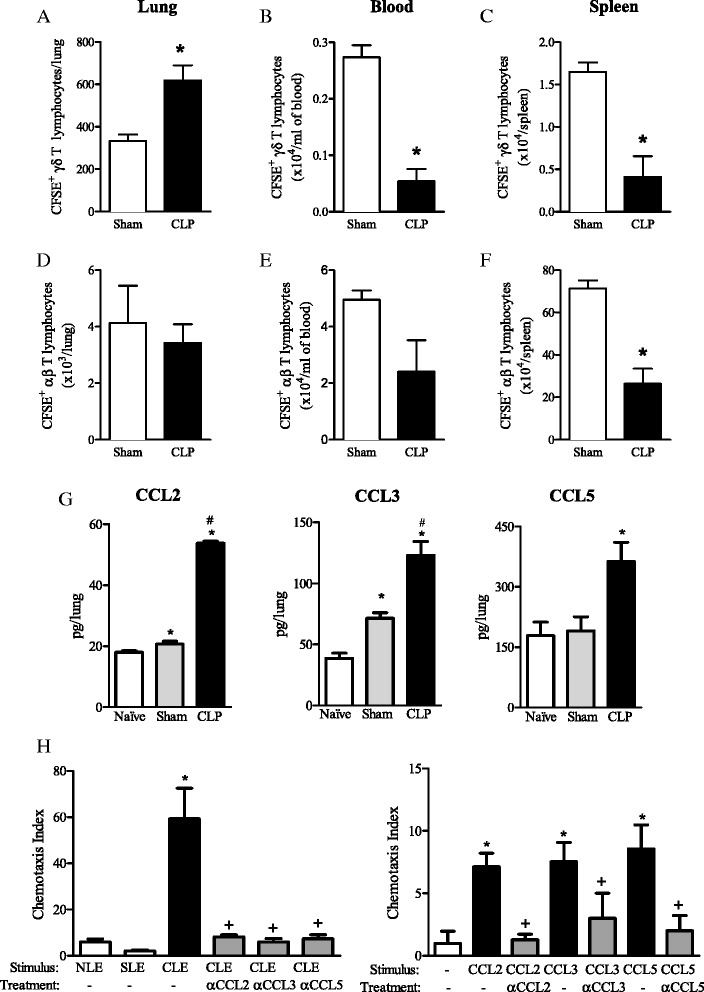
γδ T lymphocytes migrate from spleen into the lungs of CLP-operated recipient mice. T lymphocytes recovered from the spleen of naïve mice were labeled with CFSE and transferred to CLP-operated mice 3 and 8 days after surgery. Recipient animals were euthanized 10 days after surgery, and their lungs, blood and spleen were collected for γδ (**a**–**c**) and αβ (**d**–**f**) T cell analysis by flow cytometry. Quantification of CCL2, CCL3, and CCL5 levels in lung homogenates of naïve, sham and CLP C57BL/6 mice by ELISA, 7 days after surgery (**g**). γδ T cell chemotaxis towards lung homogenates from CLP mice (or towards CCL2, CCL3 and CCL5), incubated or not with neutralizing α-CCL2, α-CCL3 or α-CCL5, as described in methods (**h**). Representative results of two experiments from at least 4 animals per experimental group are expressed as mean ± SEM. Statistical differences (p < 0.05) between CLP and sham groups are indicated by (*), and between stimulated and mAb-treated groups are indicated by (+)

Ten days after surgery, CFSE^+^ γδ (but not αβ) T cells adoptively transferred from naïve mice were preferentially localized in the lungs of CLP-operated mice, when compared to blood and spleen (Fig. 3a-f). In accordance, increased levels of CCL2, CCL3 and CCL5 were detected in lung homogenates of CLP-operated mice, when compared to chemokine levels detected in naïve and sham mouse lung samples (Fig. 3g). No differences were observed in CCL25 levels between CLP- and sham-operated mice (sham 681 ± 94; CLP 753 ± 175 pg/lung). γδ T lymphocytes migrated *in vitro* towards lung homogenates obtained from CLP mice at a higher extent than towards lung homogenates from naïve or sham-operated mice. The *in vitro* neutralization of CCL2, CCL3 and CCL5 by mAbs inhibited γδ T lymphocyte chemotaxis towards the respective chemokines and lung homogenates obtained from CLP mice, suggesting that these chemokines coordinate γδ T cell *in vivo* migration into the lungs during severe sepsis (Fig. 3h).

### γδ T lymphocytes from the lungs of CLP-operated mice produce IL-17

**Fig. 4:**
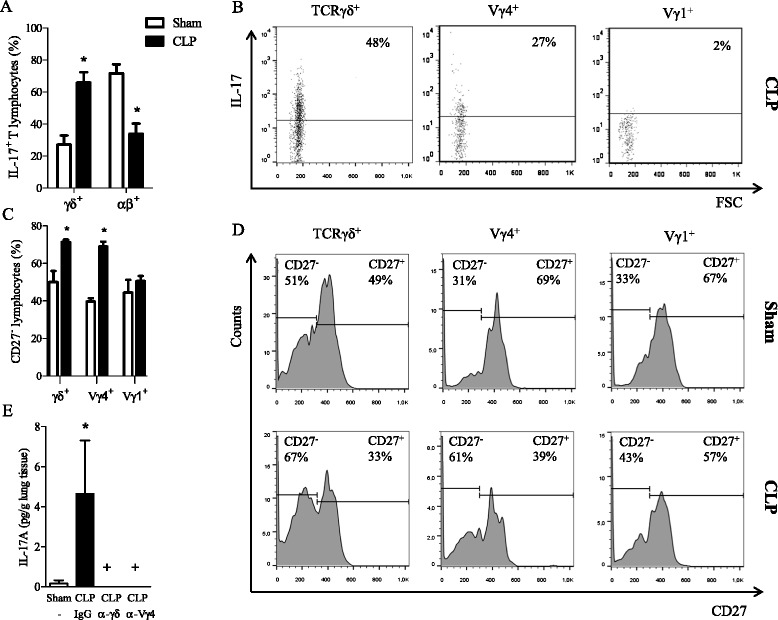
Increased IL-17 production by Vγ4 T lymphocytes in CLP mouse lungs. **a** Percentages of γδ and αβ T lymphocytes among lung IL-17^+^ T cells recovered 10 days after CLP, as determined by intracellular staining. **b** Representative dot plots of intracellular staining of IL-17^+^ within γδ, Vγ4 and Vγ1 T cells recovered from the lungs of CLP mice. **c** Percentages of CD27^-^ cells among γδ, Vγ4 and Vγ1 T cell population in the spleen recovered 3 days after CLP. **d** Representative histograms of CD27 staining of γδ, Vγ4 and Vγ1 T cells recovered from mouse spleen. **e** IL-17 quantification in lung homogenates of CLP mice treated or not with α-γδ TCR mAb, α-Vγ4 TCR mAb or control IgG, 7 days after surgery was performed by CBA. Results are expressed as mean ± SEM from at least 5 animals per group. Statistical differences (p < 0.05) between CLP and sham groups are indicated by (*), and between stimulated and mAb-treated groups are indicated by (+)

Ten days after surgery, intracellular staining revealed that the percentage of IL-17^+^ γδ T lymphocytes increased among total CD3^+^ cell population in the lungs of CLP mice, while the percentage of IL-17^+^ αβ T lymphocytes decreased after CLP, when compared to sham-operated mice (Fig. 4a). Evaluation of γδ T cell cytokine profile revealed a slight decrease in IL-10^+^ and IFN-γ^+^ γδ T lymphocytes in CLP mouse lungs, whereas no differences between IL-4^+^, IL-12^+^ or tumor necrosis factor (TNF)-α^+^ γδ T lymphocytes were detected between CLP and sham mice (Additional file 1: Figure S1A). It is noteworthy that the percentage of IL-17^+^ (but not IFN-γ^+^) γδ T lymphocytes increased upon *in vitro* restimulation with α-CD3 mAb (Additional file 1: Figure S1B-C). Representative dot plots show that IL-17 positive staining was detected among γδ^+^ and Vγ4^+^, but not among the Vγ1^+^ lymphocyte subtype recovered from the lungs of CLP mice (Fig. 4b). IL-17 production by γδ T cells is restricted to CD27^-^ cells. Accordingly, our data demonstrate that the percentage of CD27^-^ lymphocytes increased among Vγ4^+^, but not among the Vγ1^+^ lymphocytes in the spleen 3 days after CLP (Fig. 4c-d). To evaluate the implication of Vγ4 T lymphocytes in IL-17 production during sepsis, mice were treated with anti-Vγ4 mAb 1 day before CLP. Figure 4e shows that anti-Vγ4 mAb treatment decreased IL-17 production in CLP mouse lungs 7 days after surgery, in a similar extent as γδ T lymphocytes.

### Anti-Vγ4 TCR mAb treatment decreases survival rate in C57BL/6 mice subjected to severe sepsis

**Fig. 5:**
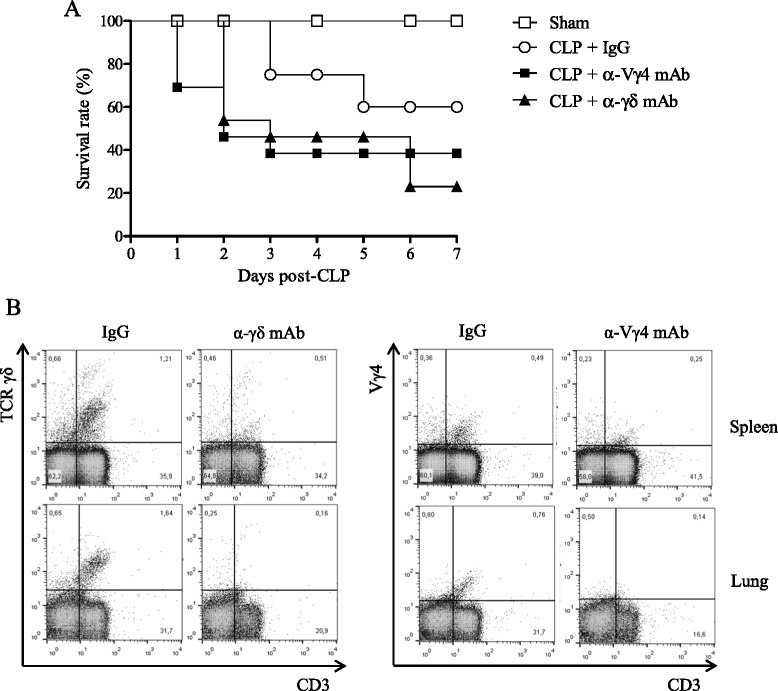
Anti-Vγ4 mAb treatment decreases survival rate of septic mice. **a** Survival rate was analyzed in CLP mice treated or not with α-γδ TCR mAb, α-Vγ4 TCR mAb or IgG up to 7 days after CLP surgery. The results are expressed as percentage of survival rate per day, from 10 mice per group. **b** Representative dot plots of γδ and Vγ4^+^ T cell frequency in spleen and lungs of treated mice, analyzed by flow cytometry

Approximately 50 % of C57BL/6 mice subjected to the experimental model of severe sepsis and antibiotic treatment died within 7 days (Fig. 5a). Anti-Vγ4 mAb treated mice that underwent CLP died in shorter periods of time, achieving 30 % of survival rate within 7 days. Worthy of note, anti-γδ mAb treatment similarly precipitated CLP mouse death, suggesting that Vγ4 T cell subset presents a protective role in septic mice. IgG isotype-treated mice showed similar survival rate than untreated mice. Figure 5b and Additional file 2: Figure S2A–B show the effectiveness of depletion by mAb administration in spleen and lungs.

## Discussion

Sepsis triggers a complex immune response that involves both innate and adaptive systems. γδ T lymphocytes represent a link between these two branches of the immune system, by coordinating the activation of different cell populations via cytokine production [27]. γδ T lymphocytes have been described as a major source of IL-17 in peritoneum and lymphoid organs during experimental sepsis, a phenomenon shown to present either beneficial or deleterious effects, depending on the experimental model [33–35, 38]. The data presented here identifies the Vγ4 subset as a dominant producer of IL-17 in the lungs of septic mice and as a central T cell population involved in host defense against sepsis.

The experimental model of severe sepsis used in the present work resulted in the accumulation of T lymphocytes in lung tissue, which were likely originated from lymphoid organs. Increased numbers of both γδ and αβ T lymphocyte subsets were detected in the lungs; however it is noteworthy that, differently from αβ T lymphocytes, γδ T cell numbers continually increased up to day 10 after CLP, mainly due to the accumulation of Vγ4 subset. The progressive accumulation of γδ T cells in the lungs of CLP-operated mice has been previously demonstrated by Hirsh and coworkers [7]; however, the presence of γδ T cell subtypes has not been described. The decrease in αβ T cell numbers observed at day 3 after CLP is in accordance with several reports in mice and humans that demonstrate a reduction in circulating CD3^+^ T lymphocytes during sepsis [39]. This reduction is explained by a massive apoptotic event of T lymphocytes, which is correlated with severity and mortality in experimental animals and patients [2, 39–41]. The fact that the percentage of CD25^+^ T lymphocytes increased among γδ T lymphocytes in the lungs at early time points after CLP (day 1) suggests that γδ (but not αβ) T lymphocytes are constantly activated in lymphoid tissues during the course of sepsis and continuously migrate towards inflamed lungs. In accordance with our data, Matsushima and co-workers [40] demonstrated the early activation of γδ T lymphocytes from peripheral blood of patients with sepsis and systemic inflammatory response syndrome. These patients presented increased percentages of peripheral CD69^+^ γδ T cells at acute time points after injuries, whereas CD69 expression by αβ T cells did not increase during a 2-week period [40]. It is noteworthy that, in our study, such early activation was also evident for Vγ4 T cell population, as observed in mouse spleen and lungs 1 day after CLP.

The selective migration of γδ T lymphocyte subsets into the tissue during inflammation is dictated by elevated levels of chemoattractant mediators in the tissue and by the expression pattern of chemokine receptors on cell surface [9, 10, 15, 16, 42, 43]. Our results suggest that γδ and Vγ4 T cell migration into the lungs of CLP mice is likely accounted by the combined *in situ* accumulation of multiple chemokines. CLP-induced lung inflammation increased tissue levels of CCL2, CCL3 and CCL5, chemokines that are known to mediate γδ T lymphocyte migration *in vivo* and *in vitro* [44–47]. Consistently, here we show that adoptively transferred γδ T cells preferentially accumulated in the lungs (rather than in blood or spleen) of recipient CLP mice. Even though CCL2, CCL3 and CCL5 are also chemoattractant for αβ T lymphocytes [48], the neutralization of these chemokines in CLP lung homogenates did not impair the chemotaxis of γδ^-^ T lymphocytes (data not shown), suggesting that these chemokines selectively dictate the migration of γδ T cells into the lungs in our model. The involvement of CCL25 in γδ T cell migration towards inflamed lungs during sepsis was also investigated by us, since CCL25 has been shown to attract IL-17^+^ γδ T cells into inflamed airways [15]. However, CCL25 was not enhanced in CLP mouse lungs (data not shown).

Our data demonstrate that the Vγ4 T lymphocyte subset predominated among the IL-17^+^ cell populations in CLP mouse lungs. In line with this, it has been demonstrated that mice lacking γδ T cells (but not αβ T cells) subjected to CLP failed to present elevated IL-17 levels in the plasma and peritoneal lavage, showing that γδ T cells are the major producers of IL-17 during experimental sepsis [33–35, 38]. It has been established that, among murine γδ T lymphocytes, IL-17 production is restricted to Vγ4 and Vγ6 subtypes [27, 49]. Consistently with our data, Vγ4 T lymphocytes comprise the major subset that migrates into the lungs and have been shown to produce IL-17 in different experimental models [10, 13, 24–30]. It is noteworthy that our supplemental data (Additional file 1: Figure S1A) demonstrate that IL-17 production by γδ T cells from CLP-mouse lungs predominated over the expression of other cytokines, including IFN-γ. These data are reinforced by the increase in the percentage of CD27^-^ γδ^+^ (and Vγ4^+^) population in CLP-mouse spleen and by the fact that, upon α-CD3 mAb *in vit*ro stimulation, these cells were enriched for IL-17 but not for IFN-γ (Additional file 1: Figure S1B–C).

Increased numbers of γδ T lymphocytes in the blood, peritoneum and lungs have been correlated with sepsis positive outcome in patients and experimental animals [8, 14, 32, 35, 50]. Indeed, mice lacking γδ T lymphocytes and subjected to CLP presented increased mortality rate and decreased survival periods [14, 32]. The protective role of γδ T lymphocytes during sepsis results from the ability of these cells to produce inflammatory mediators capable to modulate other leukocyte populations, among which IL-17 is of particular importance [35, 51, 52]. Here we show that IL-17 production in the lungs of CLP mice depends on infiltrated Vγ4 γδ T cell subset, which likely contributes to host protective immune response. Since adverse roles have been proposed for IL-17 during experimental sepsis, the effect of IL-17 in the lungs of CLP mice needs further investigation. It has been described that IL-17 derived from γδ T cells promotes epithelial repair in different tissues [53–55], suggesting that IL-17 produced by Vγ4 T cells might act on lung epithelium, promoting tissue repair and ameliorating mouse illness after CLP [33–35, 56]. IL-17 has also been associated with neutrophil influx into inflamed tissue, which can lead to either protective or harmful outcomes [35, 37, 57, 58]. Concerning lung immune response, effective bacteria clearance by neutrophils reduces the risk of lung failure [36]; however, it is well known that excessive neutrophil activation and production of myeloperoxidase (MPO) can cause tissue damage [57]. In our study, we observed increased neutrophil numbers in the lungs of CLP mice, which was significantly reduced after anti-Vγ4 mAb treatment (data not shown), suggesting neutrophil involvement in the resolutive response. The involvement of tissue-recruited neutrophils coordinated by IL-17^+^ γδ T cells in tissue repair has been demonstrated in different experimental models. γδ T cell knockout (KO) mice submitted to inflammatory insults are shown to present reduced neutrophil and MPO accumulation in the lungs, liver and cornea, which correlated with increased lesions and delayed epithelial regeneration [53–55]. Moreover, in a model of corneal epithelial abrasion, it was demonstrated that γδ T cells induced, via IL-17, the production of vascular endothelial growth factor (VEGF) by neutrophils, promoting corneal nerve regeneration [59]. Our study evidenced that IL-17^+^ Vγ4 T lymphocytes migrate into injured lungs of CLP mice, presenting a beneficial role during the course of sepsis.

## Conclusions

In the present work, we show that early-activated Vγ4 T lymphocytes continuously accumulate in inflamed lungs during the course of sepsis and that local IL-17 production depends on the tissue infiltration of this subset, which preferentially produces this cytokine. Based on our findings, we also propose that Vγ4 T lymphocytes contribute to the protective immune response of septic mice and delay mortality. Further complementary investigation concerning cellular and molecular mechanisms of Vγ4 T cell/IL-17 pathway associated with protection during sepsis is of extreme value to bring new insights to approach novel targets and therapies.

## Methods

### Cecal ligation and puncture

Polymicrobial sepsis was induced by cecal ligation and puncture (CLP) in normal fed and anesthetized (112.5 mg/kg of ketamin and 7.5 mg/kg of xylazine, i.p. Rhobifarma, Brazil) male C57BL/6 mice (18 to 20 g) provided by Oswaldo Cruz Foundation breeding unit (Rio de Janeiro, Brazil). After laparotomy (incision of 0.5–1 cm), the cecum was ligated with a cotton suture distal to the ileocecal valve to avoid bowel obstruction, and punctured nine times with a 21-gauge needle. The cecum was placed back into the abdomen and the incision was closed by a 4–0 polyamide suture. Sham-operated animals received midline laparotomies, exteriorization of the cecum with its immediate return and closure of incisions. Mice were resuscitated by a subcutaneous injection of 1 ml sterile saline solution. Mice were treated with ertapenem (Merck, Germany; 75 mg/kg, i.p.) 6, 24 and 48 h after surgery. For lung analysis, mice were euthanized in a CO_2_ chamber 1, 3, 7 and 10 days after CLP operation. For the assessment of survival rate, mice were evaluated every 12 h following CLP until death. During all experimental procedures, mice were monitored daily and those that presented impaired locomotor activity and no struggle response to sequential handling were euthanized. All experimental procedures were performed according to the Committee on Ethical Use of Laboratory Animals of Oswaldo Cruz Foundation (Fiocruz, Brazil, #L62/12).

#### Antibody treatment

Hamster anti-TCR γδ (3A10, anti-pan-δ, described by Itohara *et al*. [60]) and anti-Vγ4 (UC3-10A6, described by Dent *et al*. [61]) monoclonal antibodies (mAb) were obtained from SCID mice (Oswaldo Cruz Foundation breeding unit, Rio de Janeiro, Brazil) ascitic fluid. 3A10 preparation was further purified/concentrated by Protein G (GE Healthcare, USA) affinity chromatography while UC3 was concentrated by ammonium sulfate precipitation. Both antibody preparations were dialyzed against saline solution before use. mAbs were i.p. administered (500 μg/mice every other day for 7 days, starting 1 day before CLP). Control mice were similarly sham-treated with normal hamster serum IgG.

### Recovery of leukocytes from lung and spleen

Lung tissue samples were obtained from euthanized C57BL/6 mice at 1, 3, 7 and 10 days after CLP, macerated in RPMI 1640 medium containing collagenase type IV (250 IU/ml, 37 °C, 30 min) and centrifuged (400 *g*, 10 min). Spleens were dissected, macerated in PBS containing EDTA (10 mM, pH 7.4), and centrifuged (420 *g* for 10 min at 20 °C). Cell pellets from lung and spleen were re-suspended in 3 ml of PBS/EDTA and subjected to centrifugation on a Histopaque 1083 gradient (400 *g* for 30 min) for mononuclear cell separation.

### Flow cytometric analysis

Leukocytes were stained with the appropriate concentration of the following antibodies: PE/FITC CD3 (145–2C11), PE/FITC TCR δ chain (GL3), PE TCR β chain (H57–597), FITC Vγ4 TCR (UC3-10A6), FITC Vδ4 TCR (GL2), FITC CD25 (7D4), PE/FITC IgG1 and IgG2 isotypes (BD Pharmingen, USA) and APC Vγ1 TCR (2.11) (Biolegend, USA). For intracellular cytokine staining, cells were pre-incubated for 4 h with PMA (20 ng/ml), ionomycin (500 ng/ml) and brefeldin A (10 μg/ml) at 37 °C and 5 % CO_2_. After surface marker staining, cells were fixed, permeabilized and stained with anti-IFN-γ, anti-TNF-α, anti-IL-4, anti-IL-10, anti-IL-12 and anti-IL-17 antibodies (BD Pharmingen, USA). IgG isotypes were used as irrelevant antibodies. Cells were acquired by FACScalibur flow cytometer (Becton Dickinson, USA) and analyzed either by Cell Quest or FlowJo softwares. Counts are reported as percentage and as numbers of cells after the multiplication of the percentage of T lymphocyte population by the total number of leukocytes. Gating strategies are shown in additional files (Additional file 3: Figure S3 and Additional file 4: Figure S4).

### Adoptive transfer assay

Naïve C57BL/6 splenocytes were labeled with CFSE (Invitrogen USA, 1 μM/8×10^6^ cells) and i.v. injected (4 × 10^7^ cells, ≥ 90 % viability) into recipient mice 3 and 8 days after CLP or sham operations. Recipient mice were euthanized 10 days after adoptive transfer and their lungs were recovered for leukocyte analysis.

### Preparation of lung homogenates

Lung homogenates were prepared by homogenizing perfused whole lung tissue using a glass potter homogenizer (Kontes Glass Company, USA) in 2 ml of PBS containing cell lysis buffer (Sigma Aldrich, USA) and protease inhibitor (1 μl/ml; Sigma Aldrich, USA), at 4 °C. The homogenates were centrifuged (8400 *g* for 30 min, 4 °C) and the supernatants were filtered (0.2 μm). For chemotaxis assays, lungs were homogenized using PBS only.

### Cytokine quantification

Levels of chemokines were evaluated in lung homogenates from lungs recovered 7 days after CLP surgery by sandwich enzyme-linked immunosorbent assay (ELISA) by using matched antibody pairs from R&D (Minneapolis, MN), according to manufacturer’s instructions. IL-17 quantification was performed using the BD™ Cytometric Bead Array (CBA) mouse Th1/Th2/Th17 kit (BD Biosciences, USA), and samples were analyzed using a FACScalibur flow cytometer.

### Transwell migration assay

Spleen T lymphocytes (3 × 10^6^ in HBSS without Ca^2+^/ Mg^2+^) were placed in the upper chamber of 5.0 μm pore diameter transwell tissue culture inserts (BD Falcon, USA). Transwell inserts were placed in the individual wells of a 24-well cell culture plate containing assay buffer or lung homogenates from naïve, sham-operated and CLP-operated mice, neutralized (30 min, 37 °C) with anti-CCL2 mAb (2.5 ng/well), anti-CCL3 mAb (200 ng/well) or anti-CCL5 mAb (50 ng/well). The recombinant chemokines rmCCL2 (2.5 ng/well), rmCCL3 (4 ng/well) and rmCCL5 (4 ng/well) (R&D Systems, USA) were used as positive controls. After 2 h, the migrated cells were counted, labeled as described above, and analyzed by FACScalibur. Results are expressed as chemotactic index, generated by using the number of cells that migrated towards buffer as comparison.

### Statistical analysis

Data are reported as the mean ± SEM and were statistically evaluated by analysis of variance (ANOVA) followed by Newman-Keuls-Student test or Student’s t test. Values of p ≤ 0.05 were regarded as significant.

## Additional files

Additional file 1: Figure S1. Cytokine production by γδ T lymphocytes from the lungs of CLP-operated mice. (A) Percentage of IL-4^+^, IL-10^+^, IL-12^+^, IFN-γ^+^ and TNF-α^+^ γδ T lymphocytes obtained from the lungs of C57BL/6 mice 10 days after CLP or sham surgery. Cells were cultured with brefeldin A (10 μg/ml, 4 h), submitted to intracellular staining and analyzed by flow cytometry. Results are expressed as mean ± SEM from at least 4 animals per experimental group. (B) Percentage of IL-17^+^ and (C) IFN-γ^+^ γδ T lymphocytes within the population of splenic γδ T lymphocytes recovered 10 days after CLP, stimulated *ex-vivo* with α-CD3 mAb (5 μg/ml, 4 h), submitted to intracellular staining and analyzed by flow cytometry. Statistical differences between the CLP or α-CD3-stimulated groups and the negative control groups (p < 0.05) are indicated by (*). Gates were established after the staining with their IgG isotypes. Additional file 2: Figure S2. γδ T cell depletion induced by α-γδ mAb (3A10) administration. To certify the effectiveness of α-γδ mAb treatment, γδ TCR staining was performed in permeabilized cells recovered from C57BL/6 mouse spleens after α-γδ TCR mAb (3A10) or hamster serum IgG administration. (A) Representative dot plots of intracellular γδ TCR staining with UC7 (Southern Biotech, USA) and GL3 (Caltag, UK) mAbs in αβ^-^/B220^-^ cell population. (B) Representative histograms of intracellular γδ TCR staining (GL3 mAb) of αβ^-^/B220^-^ cells recovered from α-γδ TCR mAb (3A10) or hamster serum IgG-treated mouse, placed in culture for 48 h. Additional file 3: Figure S3. Gating strategies used for FACS analysis of γδ and αβ T lymphocytes. A lymphocyte gate (R1) was defined based on the cells’ Forward Scatter (FSC) and Side Scatter (SSC), further gated on TCRγδ^+^ (R2) or αβ^+^ (R3) lymphocytes. Additional file 4: Figure S4. Gating strategies used for FACS analysis of γδ and αβ T lymphocytes within IL-17^+^ cells. IL-17^+^ lymphocyte gate (R6) was defined and further gated on TCRγδ^+^ and αβ^+^ lymphocytes.

## Abbreviations

APC: Allophycocyanin
ARDS: Acute respiratory distress syndrome
CBA: Cytometric Bead Array
CCL: CC chemokine ligand
CD: Cluster of differentiation
CFSE: Carboxyfluorescein succinimidyl ester
CLP: Cecal ligation and puncture
CO_2_: Carbon dioxide
EDTA: Ethylenediamine tetraacetic acid
ELISA: Enzyme-linked immunosorbent assay
FACS: Fluorescence activated cell sorter
FITC: Fluorescein isothiocyanate
*g*: Gravity
IFN: Interferon
IgG: Immunoglobulin
IL: Interleukin
i.p: Intraperitoneal
i.v: Intravenously
KO: Knockout
mAb: Monoclonal antibody
min: Minute
ml: Milliliter
MPO: Myeloperoxidase
PBS: Phosphate buffered saline
PE: Phycoerythrin
PMA: Phorbol-12-myristate-13-acetate
RM: recombinant murine
RPMI: Roswell Park Memorial Institute
SEM: Standard error of the mean
TCR: T cell receptor
TNF: Tumor necrosis factor
VEGF: Vascular endothelial growth factor
