# Supplementary figures and images for: Murine IL-17+ Vγ4 T lymphocytes accumulate in the lungs and play a protective role during severe sepsis
Source: BMC Immunol. 2015 Jun 3;16:36. doi: 10.1186/s12865-015-0098-8 (PMC4451961; doi:10.1186/s12865-015-0098-8)

## Slide 1
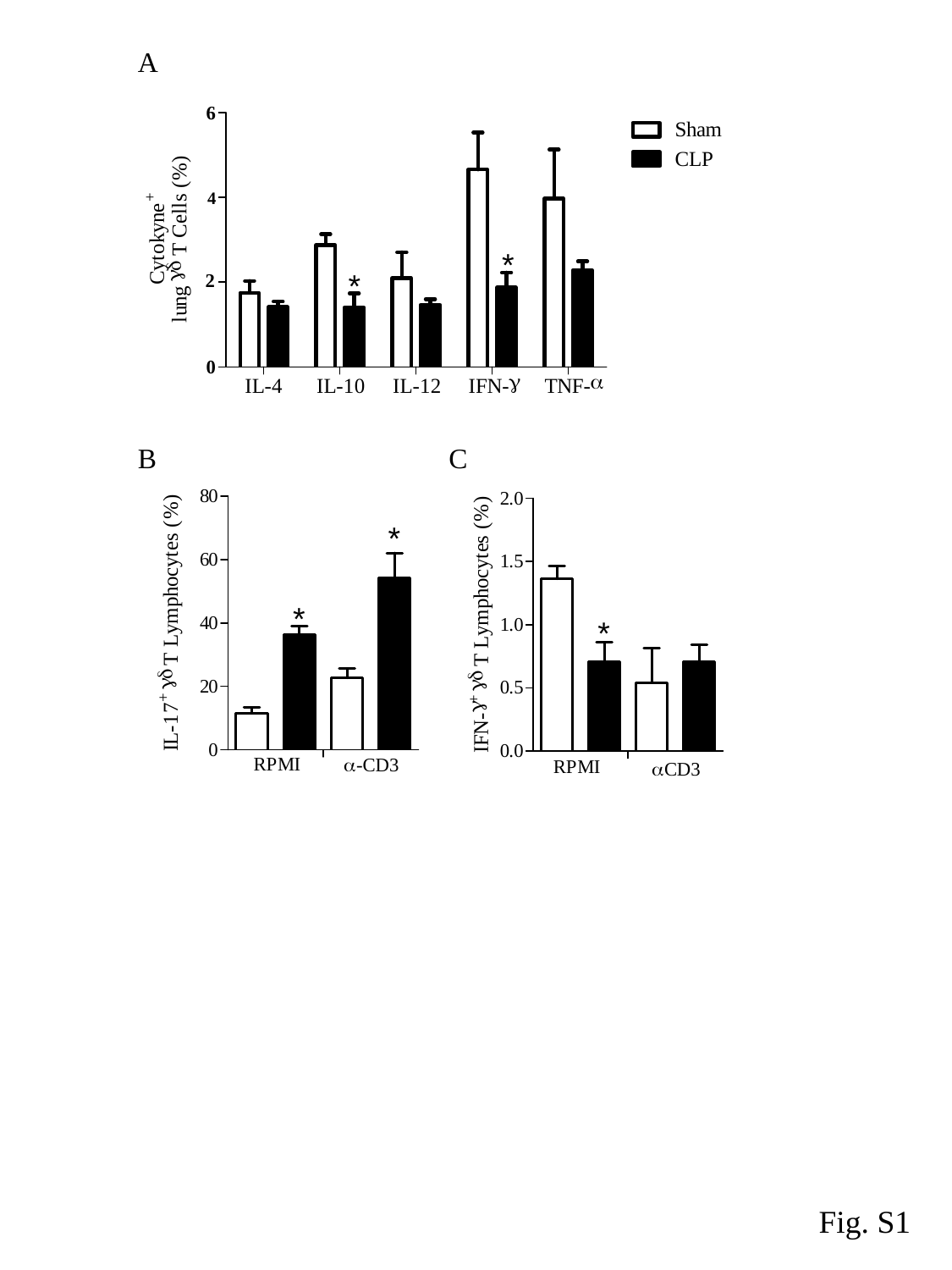

A
B
C
Fig. S1

Supplement: Additional file 1: Figure S1. — Cytokine production by γδ T lymphocytes from the lungs of CLP-operated mice. (A) Percentage of IL-4+, IL-10+, IL-12+, IFN-γ+ and TNF-α+ γδ T lymphocytes obtained from the lungs of C57BL/6 mice 10 days after CLP or sham surgery. Cells were cultured with brefeldin A (10 μg/ml, 4 h), submitted to intracellular staining and analyzed by flow cytometry. Results are expressed as mean ± SEM from at least 4 animals per experimental group. (B) Percentage of IL-17+ and (C) IFN-γ+ γδ T lymphocytes within the population of splenic γδ T lymphocytes recovered 10 days after CLP, stimulated ex-vivo with α-CD3 mAb (5 μg/ml, 4 h), submitted to intracellular staining and analyzed by flow cytometry. Statistical differences between the CLP or α-CD3-stimulated groups and the negative control groups (p < 0.05) are indicated by (*). Gates were established after the staining with their IgG isotypes. [file 12865_2015_98_MOESM1_ESM.ppt]

## Slide 1
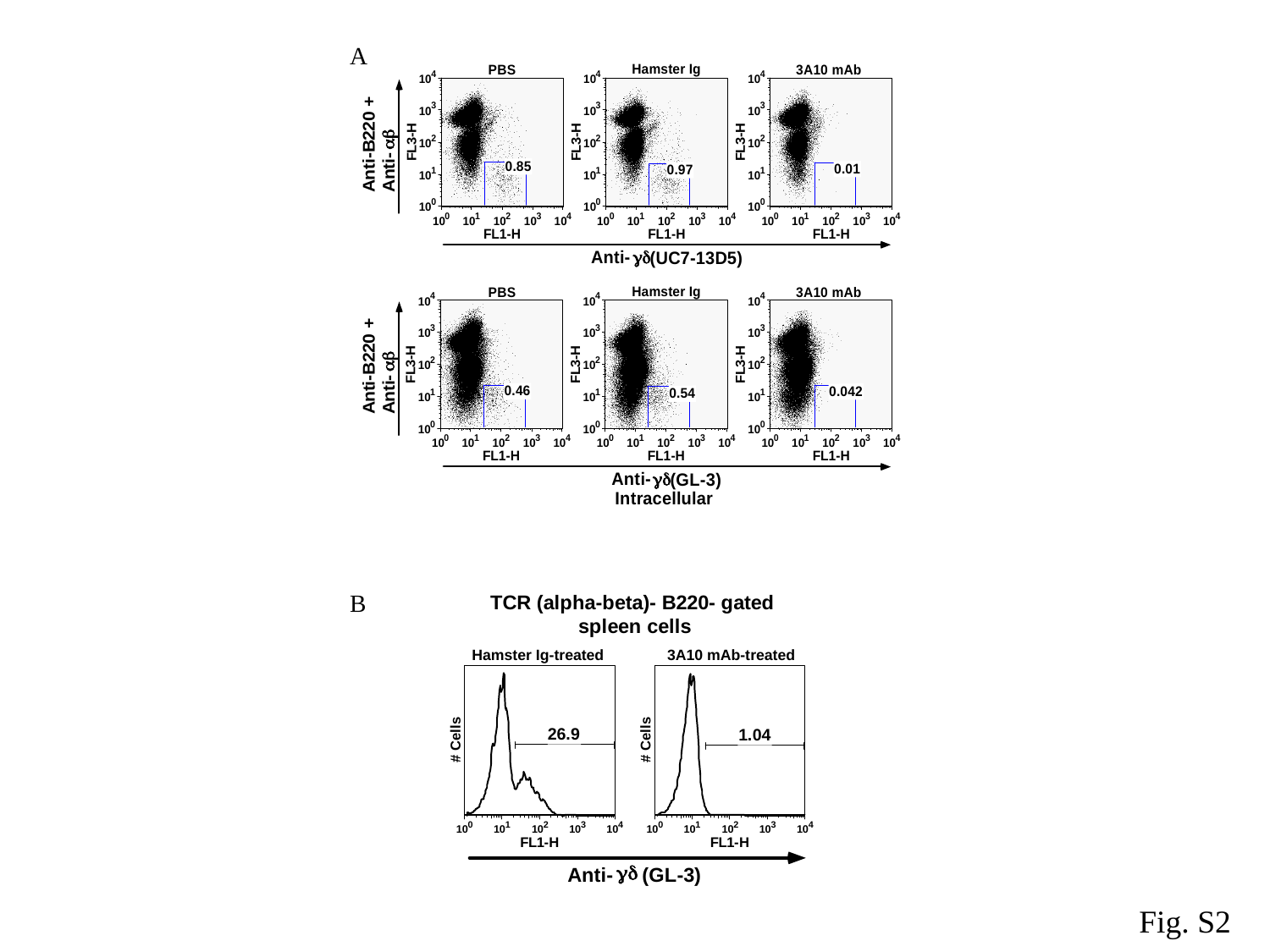

A
B
Fig. S2

Supplement: Additional file 2: Figure S2. — γδ T cell depletion induced by α-γδ mAb (3A10) administration. To certify the effectiveness of α-γδ mAb treatment, γδ TCR staining was performed in permeabilized cells recovered from C57BL/6 mouse spleens after α-γδ TCR mAb (3A10) or hamster serum IgG administration. (A) Representative dot plots of intracellular γδ TCR staining with UC7 (Southern Biotech, USA) and GL3 (Caltag, UK) mAbs in αβ-/B220- cell population. (B) Representative histograms of intracellular γδ TCR staining (GL3 mAb) of αβ-/B220- cells recovered from α-γδ TCR mAb (3A10) or hamster serum IgG-treated mouse, placed in culture for 48 h. [file 12865_2015_98_MOESM2_ESM.ppt]

## Slide 1
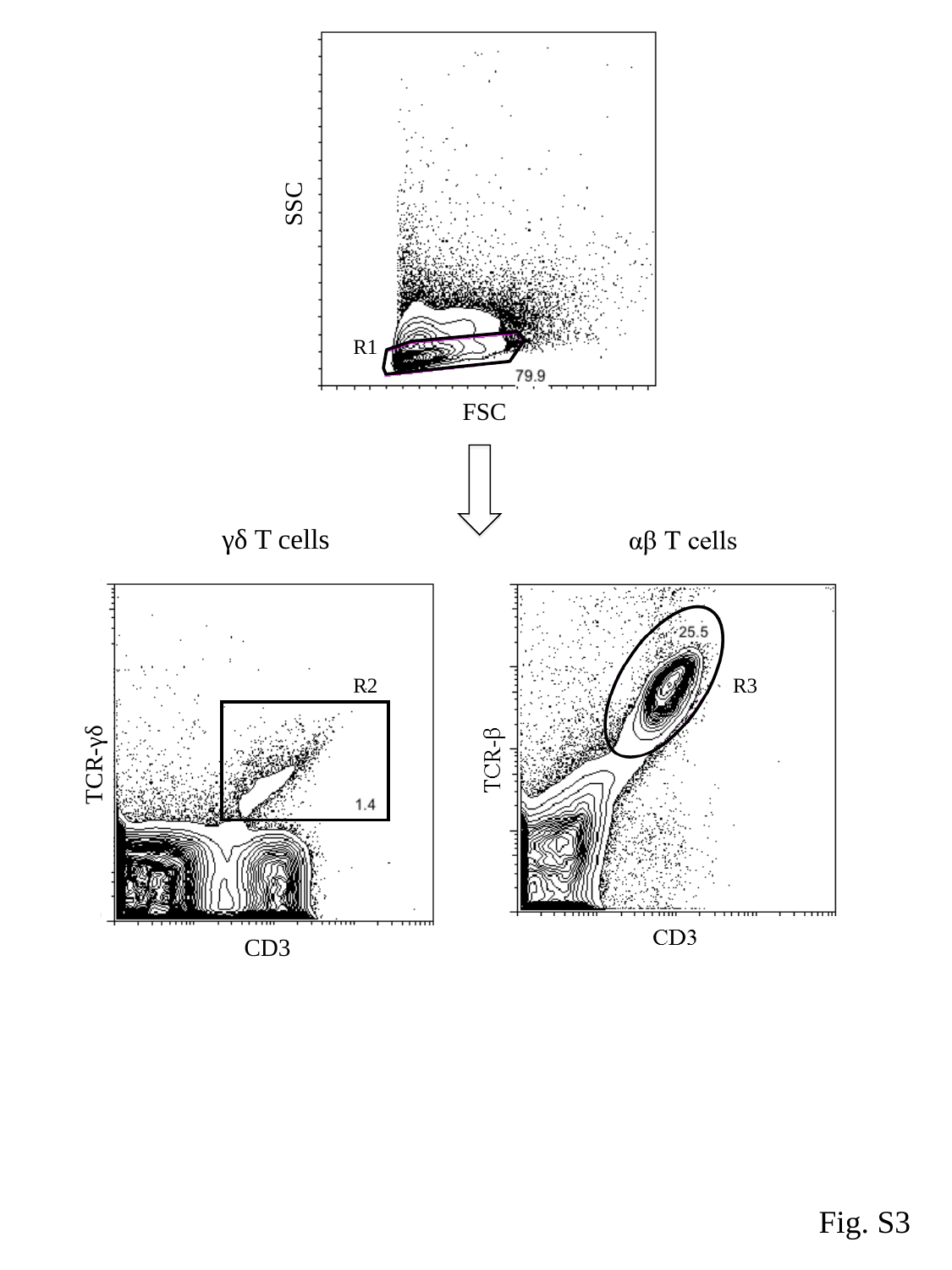

SSC
FSC
R1
γδ T cells
TCR-γδ
CD3
R2
R3
Fig. S3

Supplement: Additional file 3: Figure S3. — Gating strategies used for FACS analysis of γδ and αβ T lymphocytes. A lymphocyte gate (R1) was defined based on the cells’ Forward Scatter (FSC) and Side Scatter (SSC), further gated on TCRγδ+ (R2) or αβ+ (R3) lymphocytes. [file 12865_2015_98_MOESM3_ESM.ppt]

## Slide 1
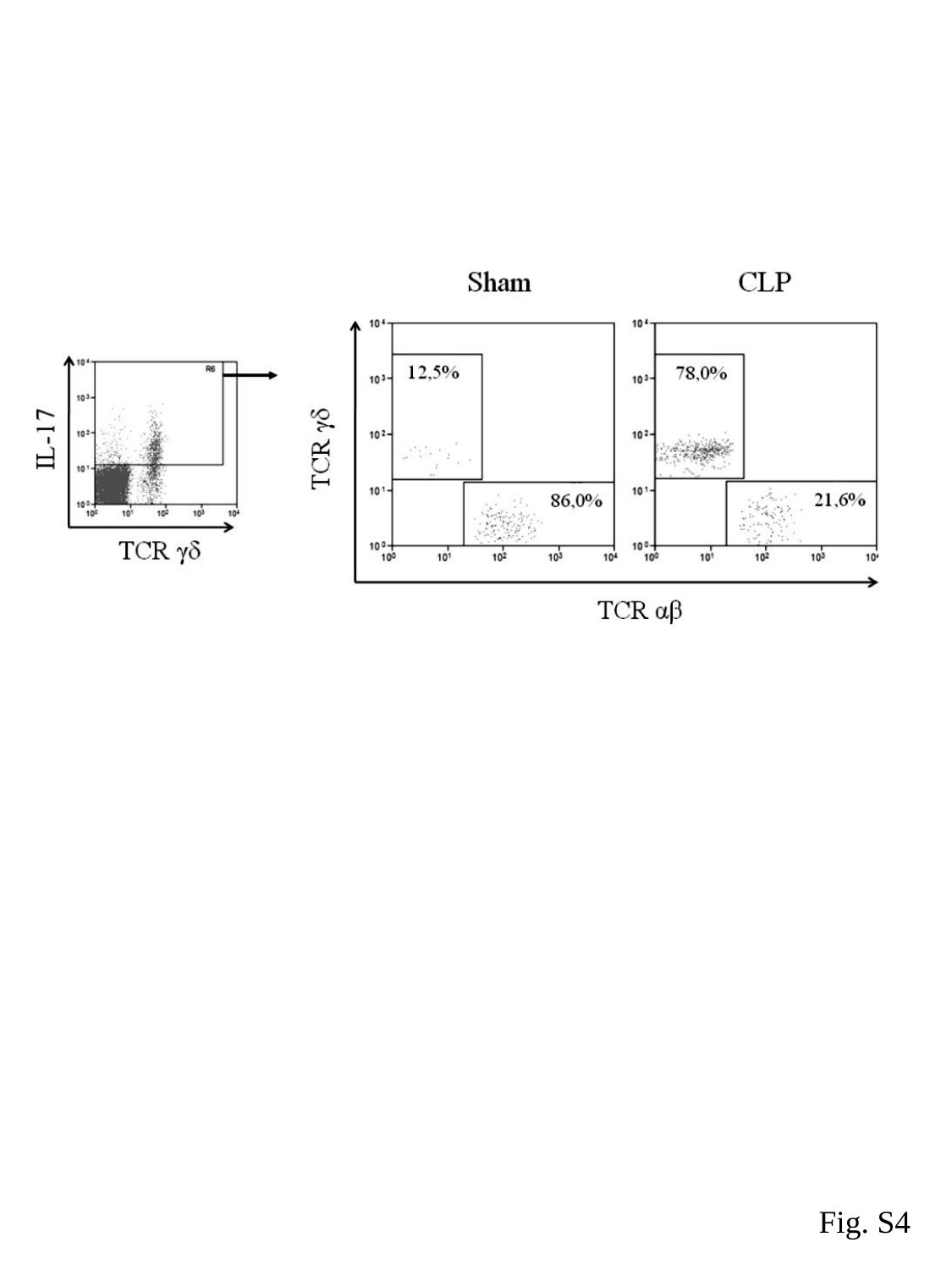

Fig. S4

Supplement: Additional file 4: Figure S4. — Gating strategies used for FACS analysis of γδ and αβ T lymphocytes within IL-17+ cells. IL-17+ lymphocyte gate (R6) was defined and further gated on TCRγδ+ and αβ+ lymphocytes. [file 12865_2015_98_MOESM4_ESM.ppt]
